# INTERPERSONAL, COMMUNITY, AND SOCIETAL STRESSORS MEDIATE BLACK–WHITE MEMORY DISPARITIES

**DOI:** 10.1093/geroni/igad104.0174

**Published:** 2023-12-21

**Authors:** Emily Morris, Jordan Palms, Ji Hyun Lee, Ketlyne Sol, Kiana Scambray, Lindsay Kobayashi, Jacqui Smith, Laura Zahodne

**Affiliations:** University of Michigan, Ann Arbor, Michigan, United States; University of Michigan, Ann Arbor, Michigan, United States; Montana State University, Bozeman, Montana, United States; University of Michigan, Ann Arbor, Michigan, United States; University of Michigan, Ann Arbor, Michigan, United States; University of Michigan School of Public Health, Ann Arbor, Michigan, United States; University of Michigan, Ann Arbor, Michigan, United States; University of Michigan, Ann Arbor, Michigan, United States

## Abstract

Psychosocial factors may contribute to racial disparities in cognitive aging beyond socioeconomic status. Contextual stressors at multiple ecological levels, which may be related to structural racism, disproportionately affect Black older adults in the U.S. and may explain increased dementia risk. This study examined the extent to which interpersonal, community, and societal level stressors independently explained Black-White disparities in memory at baseline and over time. The sample included 16,890 older Black and White adults (Mage=67.98, 22% Black) from the US Health and Retirement Study who completed psychosocial questionnaires and a word list memory task. Interpersonal, community, and societal stressors were operationalized as self-reported everyday discrimination, neighborhood physical disorder, and subjective societal status, respectively. Latent growth curves modeled memory performance over six years. Stressors were modeled simultaneously and allowed to correlate. Covariates included age, sex, education, wealth, and questionnaire year. Compared to White older adults, Black older adults experienced higher discrimination (b = -.004, SE=.001, p<.001), greater neighborhood physical disorder (b = -.012, SE=.002, p<.001), and lower perceived societal status (b = -.003, SE=.001, p=.001), each of which independently mediated the association between Black race and worse initial memory. Together, these stressors explained 11% of the racial disparity in initial memory. There was no racial disparity in memory decline. Individual experiences of stressors across multiple contextual levels contribute to racial disparities in memory, providing evidence that structural racism negatively influences dementia risk among Black older adults. Future research should consider protective factors that buffer against negative impacts of structural racism on health.

